# Adaptive Control for Virtual Synchronous Generator Parameters Based on Soft Actor Critic

**DOI:** 10.3390/s24072035

**Published:** 2024-03-22

**Authors:** Chuang Lu, Xiangtao Zhuan

**Affiliations:** School of Electrical Engineering and Automation, Wuhan University, Wuhan 430072, China; chuanglu@whu.edu.cn

**Keywords:** reinforcement learning, soft actor critic, virtual synchronous generator, virtual inertia, damping coefficient, adaptive control

## Abstract

This paper introduces a model-free optimization method based on reinforcement learning (RL) aimed at resolving the issues of active power and frequency oscillations present in a traditional virtual synchronous generator (VSG). The RL agent utilizes the active power and frequency response of the VSG as state information inputs and generates actions to adjust the virtual inertia and damping coefficients for an optimal response. Distinctively, this study incorporates a setting-time term into the reward function design, alongside power and frequency deviations, to avoid prolonged system transients due to over-optimization. The soft actor critic (SAC) algorithm is utilized to determine the optimal strategy. SAC, being model-free with fast convergence, avoids policy overestimation bias, thus achieving superior convergence results. Finally, the proposed method is validated through MATLAB/Simulink simulation. Compared to other approaches, this method more effectively suppresses oscillations in active power and frequency and significantly reduces the setting time.

## 1. Introduction

As environmental pollution and energy shortages have become increasingly serious, renewable energy power generation technologies represented by wind power and photovoltaics have garnered significant attention [1]. Renewable energy sources are generally connected to the power grid through power electronic converters, which lack the inertia and damping characteristics inherent in a traditional synchronous generator (SG). Consequently, the substantial integration of renewable energy poses significant challenges to the operational, security, and stability aspects of the power grid [2,3,4]. To address this problem, some researchers have introduced virtual synchronous generator (VSG) control technology. The VSG emulates the rotor motion equation of an SG, providing the inverter with inertia characteristics that can enhance the stability of the power grid [5]. Nevertheless, despite the VSG introducing inertia, it also inherits the low-frequency oscillation issue observed in SG [6].

It is worth noting that the virtual synchronous generator is a control algorithm executed by software, and its parameters are flexible and adjustable. Consequently, some researchers enhance the dynamic response of the VSG by adaptively adjusting parameters such as virtual inertia or the damping coefficient. In [7], an adaptive virtual inertia control strategy based on improved bang-bang control is proposed, which effectively suppresses frequency oscillation, but it allows for altering the control signal only within a constrained range of discrete values, resulting in significant jitter when it encounters interference. Ref. [8] analyzes the impact of virtual inertia on the dynamic response of VSG and designed a continuous adaptive function of virtual inertia to make the control signal smoother, but it does not consider the damping coefficient. In order to achieve better optimization results, ref. [9] designs double adaptive functions for the virtual inertia and damping coefficient. However, linear functions cannot accurately express the changing rules of parameters. Ref. [10] uses a fuzzy controller to make up for the shortcomings of linear functions. Ref. [11] also adds the adaptive control of droop gain, which provides better state of charge (SOC) maintenance capabilities for the energy storage system and avoids additional oscillations under low power consumption. The above methods are all designed based on expert experience and therefore lack objectivity and universal applicability. As the external environment and technology change rapidly, the experience of experts may quickly become outdated. Updating the knowledge and rules in expert systems is a complex and time-consuming process.

To avoid relying too much on expert knowledge, some researchers use heuristic algorithms to optimize the parameters of VSG. For example, in [12], the particle Swarm Optimization (PSO) is used to optimize the parameters and virtual impedance of VSG, which improves the stability of the microgrid and reduces the current overshoot. Additionally, ref. [13] employs a multi-objective genetic algorithm to select optimal parameters of VSG, leading to reduced maximum frequency deviation and shortened setting time. In the work of [14], a control in the virtual inertia control loop based on a proportionl-integral (PI) controller is designed, and the authors use the Manta Ray Foraging Optimization (MRFO) algorithm to optimize the PI controller. Moreover, ref. [15] takes into account the uncertainty of VSG dynamics and system inertia and uses the whale optimization algorithm to optimize and improve the virtual inertia loop. In general, heuristic algorithms can not only achieve different optimization effects according to the objective functions, but they also flexibly handle various constraints. Compared with methods based on expert experience, they have wider applicability. Nonetheless, heuristics still have their limitations. The iterative process of heuristic algorithms requires a systematic mathematical model, but power systems usually have complex topological structures and many uncertain factors, so the establishment of accurate power system models is a very arduous task. Furthermore, heuristic algorithms are resource-intensive, requiring significant time and memory, necessitating advanced hardware capabilities, and presenting challenges in satisfying the real-time demands of power system control.

Reinforcement learning (RL) is a machine learning paradigm where an agent iteratively interacts with its environment, making decisions based on feedback from past actions, with the ultimate goal of maximizing its cumulative reward over time [16,17,18]. In [19], the work employs a Q-learning algorithm to fine-tune the controller parameters of the VSG during the frequency control process. However, as a method characterized by discrete states and actions, Q-learning relies on a Q-table to archive the Q-value for every possible state–action combination. Consequently, with the expansion in the number of state and action pairs, the efficiency of Q-learning is prone to diminishing. With the evolution of deep learning, the fusion of RL with neural networks, known as deep reinforcement learning (DRL), has delivered outstanding outcomes. For example, Deep Q-Network (DQN) adopts neural networks to approximate the Q-value, enabling it to manage complex, high-dimensional environments and significantly lower the memory demands associated with Q-learning [20]. Proximal Policy Optimization (PPO) is a policy-based reinforcement learning algorithm that optimizes the policy itself and can output a continuous distribution of action probabilities, making it particularly suitable for addressing problems with continuous action spaces [21]. As for VSG, ref. [22] introduced an adaptive tuning approach for VSG’s parameters using the DQN algorithm. However, the discrete action set of the DQN algorithm poses a limitation for adjusting VSG parameters within a continuous value range. In [23], the authors use the Deep Deterministic Policy Gradient (DDPG) algorithm to adjust the virtual inertia and damping coefficient of the islanded VSG. DDPG, being a reinforcement learning algorithm based on the actor–critic framework, operates in continuous state and action spaces [24,25,26]. Nevertheless, it is well-documented that DDPG is susceptible to overestimation bias, potentially resulting in suboptimal control policies. Twin Delayed Deep Deterministic Policy Gradient (TD3) is an extension of DDPG, that can reduce overestimation bias and enhance the stability and efficiency of learning by using twin Q-networks and delayed policy updates [27].

Based on the above inspiration, this paper investigates the optimal control issue of VSG in a model-free context and employs the Soft Actor Critic (SAC) algorithm to identify the optimal strategy. SAC incorporates entropy regularization, which endows it with superior exploration capabilities, enhanced sample efficiency, and a more stable and accelerated training process compared to DDPG. Considering the over-optimization issue in traditional VSG optimization methods, a setting time penalty has been incorporated into the reward function to prevent excessive damping of frequency fluctuations, which could result in prolonged adjustment periods and compromise system stability. The main contributions of this work are as follows:

1. The optimal adaptive control problem of VSG is transformed into an RL task, thereby obviating the necessity for intricate mathematical models and expert knowledge. Subsequently, the state-of-the-art SAC algorithm is employed to train the agent, enabling it to discover the optimal strategy.
2. The traditional optimization objective of VSG focuses solely on mitigating active power and frequency fluctuations, overlooking the optimization of the system’s transient response time. In the reward function design, this paper introduces an adjustment time component, motivating the agent to refine the strategy further for enhanced system performance.

The rest of this paper is organized as follows. Section 2 delves into the fundamental principles of VSG, formulates the optimization issue as a multi-objective function, and subsequently elucidates the principles behind parameter selection. Section 3 converts the optimization problem of the VSG into an RL task, detailing the design of the reward function and the configuration of the agent. Section 4 carries out simulation experiments in MATLAB/Simulink to validate the effectiveness of the proposed method. Finally, Section 5 concludes this paper, outlining prospects for future work.

## 2. System Model and Problem Formulation

### 2.1. VSG Control Principle

**Figure 1:**
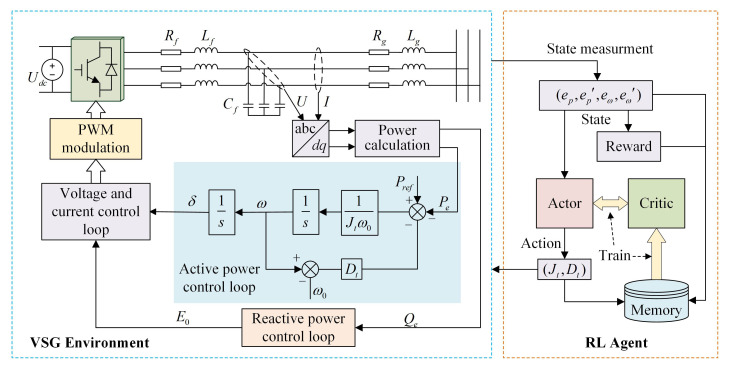
VSG control framework based on reinforcement learning.

Figure 1 shows the schematic diagram of VSG control with RL, where $R_{f}$, $L_{f}$ and $C_{f}$ represent the resistance, inductance and capacitance of the filter circuit; $R_{g}$ and Lg represent the resistance and inductance of the output line; $U_{dc}$ is the DC terminal voltage; *U* and *I* are the inverter output voltage and current. As shown in Figure 1, VSG operates as an outer loop control, primarily comprising an active power control loop (APL) and a reactive power control loop (RPL). The APL achieves control of active power and frequency by simulating the primary frequency regulation characteristics of synchronous generators; the RPL employs droop control or a PI controller to regulate reactive power and voltage; and the voltage and current control loop employs a dual closed-loop PI controller to achieve zero steady-state error tracking of the inverter’s output voltage and current. The core function of VSG lies in the APL, which provides inertia and damping to the inverter by simulating the rotor motion equation of SG [28,29]. Therefore, the purpose of this paper is to improve the APL. The rotor motion equation is described as follows:

(1) $$J\frac{d\omega }{dt}=\frac{P_{ref}}{\omega_{0}}-\frac{P_{e}}{\omega_{0}}-D\left(\omega -\omega_{0}\right),$$

where $P_{ref}$ and $P_{e}$ represent the reference active power and output active power, respectively; ω and $\omega_{0}$ represent the rotor angular velocity and rated angular velocity, respectively; and *J* and *D* are the virtual inertia and damping coefficient.

When the circuit impedance exhibits inductive behavior (or a virtual impedance is employed otherwise), the APL and RPL are approximately decoupled. In accordance with the power flow calculation equation, the active power computation formula under these conditions is delineated as follows:

(2) $$P_{e}=\frac{3E_{0}U_{g}}{Z}\sin\delta$$

where $E_{0}$ and Ug represent the effective value of inverter and grid phase voltages, respectively; *Z* is the total effective reactance of the line; and δ is the power angle of VSG, which can be given by

(3) $$\delta =\int \left(\omega -\omega_{g}\right)dt$$

where $\omega_{g}$ is the grid frequency. Typically, the value of power angle δ is small. For analytical simplification, it is approximated by setting sinδ≈δ.

To conduct a more in-depth analysis of the response characteristics of the VSG and examine the impact of virtual inertia and damping coefficients on system stability, a small signal model can be constructed based on Equations (1)–(3):

(4) $$\left\{\begin{array}{cc} J\frac{d\Delta \omega }{dt} & =\frac{\Delta P_{ref}}{\omega_{0}}-\frac{\Delta P_{e}}{\omega_{0}}-D\Delta \omega \\ \Delta \delta & =\int \Delta \omega dt\\ \Delta P_{e} & =\frac{3E_{0}U_{g}}{Z}\Delta \delta \end{array}\right.$$

where Δω, $\Delta P_{ref}$, $\Delta P_{e}$, Δδ represent the change in VSG angular frequency, reference active power, output active power and power angle, respectively. According to Equation (4), the transfer function of the active power outer loop can be expressed as follows:

(5) $$G_{p}\left(s\right)=\frac{\Delta P_{e}}{\Delta P_{ref}}=\frac{K_{p}}{J\omega_{0}s^{2}+D\omega_{0}s+K_{p}}$$

(6) $$G_{\omega }\left(s\right)=\frac{\Delta \omega }{\Delta P_{ref}}=\frac{s}{J\omega_{0}s^{2}+D\omega_{0}s+K_{p}}$$

where $K_{p}=\frac{3E_{0}U_{g}}{Z}$. $G_{p}\left(s\right)$ and $G_{\omega }\left(s\right)$ represent the transfer function of frequency response and active power response, respectively.

**Figure 2:**
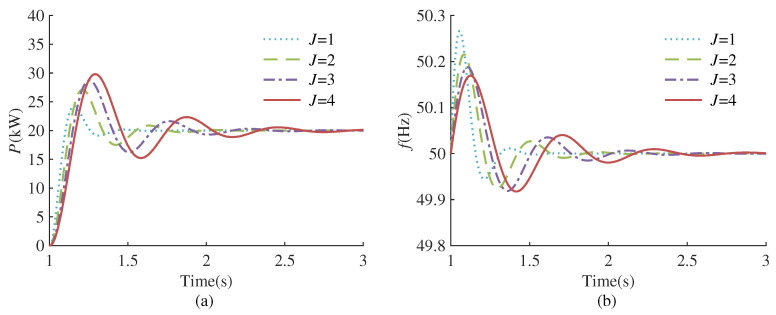
Dynamic response of VSG under different virtual inertia. (**a**) Active power response. (**b**) Frequency response.

**Figure 3:**
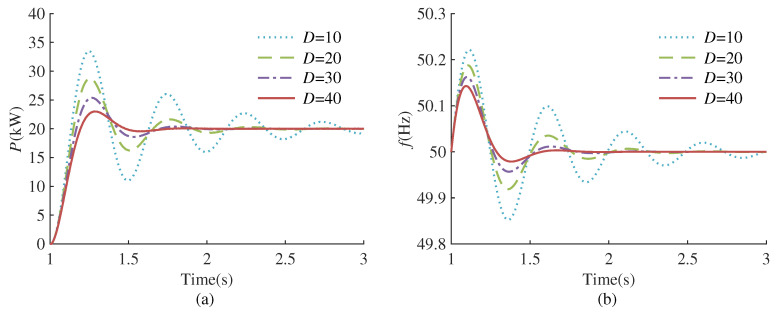
Dynamic response of VSG under different damping coefficients. (**a**) Active power response. (**b**) Frequency response.

According to the transfer function (5) and (6), it can be seen that the dynamic response of VSG is affected by virtual inertia and the damping coefficient. Figure 2 and Figure 3 plot the response curves of VSG frequency and output active power under different virtual inertia and damping coefficients, respectively.

As depicted in Figure 2, with a fixed damping coefficient, the reduction in virtual inertia leads to a decrease in active power overshoot and a shorter setting time. However, this reduction is accompanied by an increase in frequency deviation. According to Figure 3, when the virtual inertia is fixed, as the damping coefficient increases, the active power overshoot decreases, the setting time is shortened, and the frequency deviation also becomes smaller. Nevertheless, a progressive increment in the damping coefficient may transition the system from an under-damped to an over-damped state, thereby prolonging the setting time and negatively impacting the system’s stability. The analysis indicates that the virtual inertia governs the oscillation frequency during the dynamic response of VSG, while the damping coefficient determines the attenuation rate of the oscillation process. A solitary adjustment of either the virtual inertia or damping coefficient fails to attain a comprehensive optimization of active power, frequency, and the overall system response speed. Consequently, this paper employs RL algorithms to enhance VSG control and pursue the optimal control strategy. The entire control framework is depicted in Figure 1. In each control cycle, the RL agent receives the state information and reward signals from the environment, subsequently generating actions via the actor network. The actions are set to the virtual inertia $J_{t}$ and damping coefficient $D_{t}$ at the current instance. During training, the agent continuously interacts with the environment and improves the strategy through the rewards obtained, thereby finding the optimal strategy.

### 2.2. Objective Function

This paper describes the oscillation suppression process of VSG as a multi-objective optimization problem. According to the optimization objective, the objective function can be described as follows:

(7) $$\left\{\begin{array}{cc} \min E & =\tau_{1}f_{p}+\tau_{2}f_{\omega }+\tau_{3}f_{T}\\ f_{p} & =\sum_{k=0}^{N}{\left(P_{e}\left(k\right)-P_{ref}\left(k\right)\right)}^{2}\\ f_{\omega } & =\sum_{k=0}^{N}{\left(\omega \left(k\right)-\omega_{0}\left(k\right)\right)}^{2}\\ f_{T} & =T_{s} \end{array}\right.$$

where *N* is the total steps; $f_{p}$, $f_{\omega }$ and $f_{T}$ represent active power deviation cost, frequency deviation cost and setting time cost, and $\tau_{1}$, $\tau_{2}$ and $\tau_{3}$ are the corresponding weight coefficients, respectively; and $T_{s}$ represent the setting time. $f_{p}$ and $f_{\omega }$ contribute to the suppression of active power and frequency oscillations; however, excessive oscillation suppression might induce sluggish changes in power and frequency, consequently resulting in longer settling times and impacting system stability. To address this concern, we introduce a setting time cost $f_{T}$ into the objective function.

### 2.3. Constraint Conditions

The active power loop of the VSG, as described by Equation (5), conforms to the characteristics of a typical second-order system. The natural oscillation angular frequency, denoted as $\omega_{n}$, and the damping ratio, denoted as ζ, can be calculated as follows:

(8) $$\left\{\begin{array}{cc} \omega_{n} & =\sqrt{\frac{K_{p}}{J\omega_{0}}}\\ \zeta & =\frac{D}{2}\sqrt{\frac{\omega_{0}}{JK_{p}}} \end{array}\right.$$

To guarantee the stability of the system, it is essential to restrict the damping ratio of the second-order system. In the case of underdamping in a second-order system, the relationship between overshoot σ% and damping ratio ζ is expressed as follows:

(9) $$\sigma \%=\exp(-\frac{\pi \zeta }{\sqrt{1-\zeta^{2}}})\times 100\%$$

Derived from Equation (9), an excessively small damping ratio can induce substantial power overshoot, potentially causing damage to power electronic devices. Conversely, an excessively large damping ratio may result in an extended system settling time, which is detrimental to system stability. Therefore, the selection of damping ratio should not be too large or too small. In this paper, the chosen range for the damping ratio is 0.6≤ζ≤1.2. In accordance with the damping ratio range, the constraints for *J* and *D* can be determined as follows:

(10) $$0.6\leq \frac{D}{2}\sqrt{\frac{\omega_{0}}{JK_{p}}}\leq 1.2$$

The relationship between the damping coefficient, frequency deviation and power deviation is give by

(11) $$D=\frac{\Delta P}{\omega_{0}\Delta \omega }$$

In adherence with the EN50438 standard [30] for renewable energy grid connection, a 1 Hz frequency change in the grid corresponds to a change in the output active power of the inverter in the range of 40% to 100% of the rated capacity [31]. This study establishes the rated capacity of the inverter at 100 kW and with a stipulated maximum allowable frequency deviation of 1 Hz. According to Equation (11), the range of values for the damping coefficient *D* can be obtained as follows:

(12) 20≤D≤50

In order to meet the stability margin of the system, it is crucial that the characteristic roots of the system do not approach the imaginary axis too closely. Therefore, the real part of the system characteristic roots must adhere to the following constraints:

(13) Re(s)=−ωnζ=−D2J≤Re(s)max

**Figure 4:**
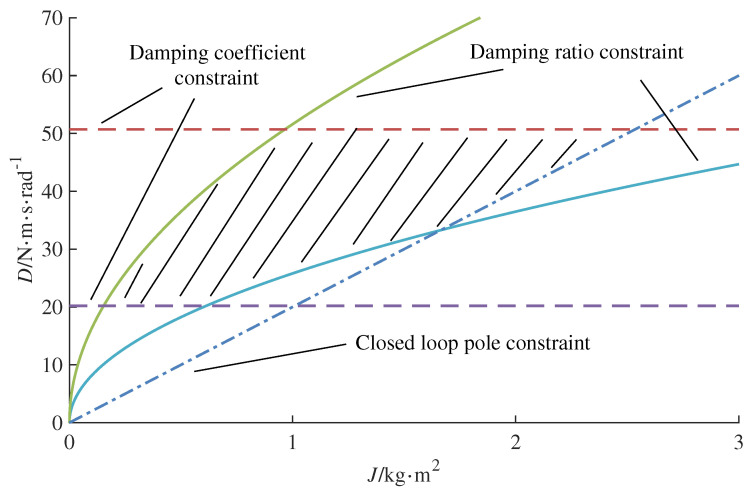
The value range of virtual inertia and damping coefficient.

In consideration of the grid capacity connected to the inverter, the range of values for Re(x)max is specified as [−10,−5]. This study adopts Re(x)max=−10. According to Equations (10), (12) and (13), the range of values for *J* and *D* is graphically depicted in a two-dimensional plane, as shown in Figure 4. The shaded area within the plot indicates the parameter range.

## 3. Transformation and Solution

### 3.1. Markov Decision Model

The parameter adjustment process of VSG can be regarded as a sequential decision-making problem. Furthermore, it satisfies the Markov property, that is, the state of the system at the next moment is only related to the state of the current moment and has nothing to do with the past state. Consequently, the adjustment of virtual inertia and damping coefficient can be reformulated as a Markov Decision Process (MDP) and addressed through a reinforcement learning algorithm. An MDP can be succinctly described by a quintuple 〈S,A,p,γ,R〉. Where S is the set of state space, A is the set of action space, *p* is the transition matrix, indicating the probability of taking action $a_{t}$ in state $s_{t}$ and transitioning to the subsequent state $s_{t+1}$, γ is the discount factor denoting the significance of future rewards in relation to current rewards (typically ranging from 0 to 1), and *R* stands for the reward function representing the feedback provided by the environment after taking action $a_{t}$ in state $s_{t}$ [32,33].

#### 3.1.1. State Space

The primary features of the dynamic response in the VSG active power loop are manifested in the responses of active power and frequency. To comprehensively depict this response process, the selection of state space is as follows:

(14) $$\left\{\begin{array}{cc} s_{t} & =\{e_{p},{e_{p}}^{\prime },e_{\omega },{e_{\omega }}^{\prime }\}\\ e_{p} & =\frac{P_{pref}-P_{e}}{P_{*}}\\ e_{\omega } & =\frac{\omega -\omega_{g}}{\omega_{*}} \end{array}\right.$$

where $e_{p}$, $e_{\omega }$, $P_{*}$ and $\omega_{*}$ represent the normalized active power deviation, normalized frequency deviation, active power normalization coefficient and frequency normalization coefficient, respectively; ${e_{p}}^{\prime }$ and ${e_{\omega }}^{\prime }$ represent the change rates of $e_{p}$ and $e_{\omega }$, respectively.

#### 3.1.2. Action Space

In this study, the control variables are the virtual inertia and damping coefficient of the VSG. Therefore, the action space is defined as the change in virtual inertia and damping coefficient and can be expressed as follows:

(15) $$a_{t}=\left\{\Delta J_{t},\Delta D_{t}\right\}$$

where $\Delta J_{t}$ and $\Delta D_{t}$ represent the changes in virtual inertia and damping coefficient, respectively. Thus, the real virtual inertia $J_{t}$ and damping coefficient $D_{t}$ can be obtained by

(16) $$\left\{\begin{array}{cc} J_{t} & =J_{0}+\Delta J_{t}\\ D_{t} & =D_{0}+\Delta D_{t} \end{array}\right.$$

where $J_{0}$ and $D_{0}$ represent the initial values of virtual inertia and damping coefficient, respectively.

#### 3.1.3. Reward Function

The objective of RL is to maximize the cumulative discounted reward, rendering the reward function a crucial element in steering the agent towards learning the optimal strategy. Existing adaptive control approaches for VSG typically focus on optimizing the oscillations in active power and frequency. However, suppressing oscillations might prolong the setting time and negatively influence the stability of the system. Consequently, this paper incorporates active power, frequency and setting time into the reward function design to address these considerations. The design methodology is as follows:

Active power deviation reward function $r_{1}$: In the post-disturbance recovery phase, the active power fluctuates around a reference value. The objective of this article is to minimize the oscillation amplitude and duration as much as possible, hence the reward function for active power is designed as follows:

(17) $$r_{1}=-c_{1}{e_{p}}^{2}$$

where $c_{1}$ represents the weight coefficient.

Frequency deviation reward function $r_{2}$: In practical scenarios, the power system necessitates that the grid frequency operates around the rated frequency, with minimal deviation being ideal. Consequently, the frequency reward function designed in this paper is structured as follows:

(18) $$r_{2}=-c_{2}{e_{\omega }}^{2}$$

where $c_{2}$ represents the weight coefficient.

Setting time reward function $r_{3}$: To prevent an excessive setting time, we incorporate the setting time as part of the reward function. Since the system can only ascertain the total settling time upon reaching a steady state, with only the last step receiving the settling time reward, this approach is deemed unreasonable. This paper adopts the following method to design a settling time reward function: for each non-steady state, a constant term penalty is applied as follows:

(19) $$r_{3}=-c_{3}$$

where $c_{3}$ represents the penalty coefficient.

Terminal reward function $r_{4}$. When the active power and frequency reach a steady state, further adjustments to the virtual inertia and damping coefficient will not alter the system state. Consequently, the sampling data in the steady state hold no value in enhancing the strategy and are considered to be an invalid sample. To improve sample efficiency, when a steady state is reached, the training for that episode is promptly terminated, and a new episode begins. To encourage the agent to approach the steady state, a positive constant reward is given when the system state reaches stability:

(20) $$r_{4}=\left\{\begin{array}{cc} M & s_{t}=s_{end}\\ 0 & s_{t}\neq s_{end} \end{array}\right.$$

where *M* is a state-independent constant, and $s_{end}$ represents the stable state. By summing Equations (17)–(20), the final reward function can be expressed as follows:

(21) $$R=-c_{1}{e_{p}}^{2}-c_{2}{e_{\omega }}^{2}-c_{3}+\left\{\begin{array}{cc} M & s_{t}=s_{\mathrm{end}}\\ 0 & s_{t}\neq s_{\mathrm{end}} \end{array}\right.$$

The roles of $r_{1}$ and $r_{2}$ is to suppress the oscillation of active power and frequency, and the function of $r_{3}$ is to reduce the setting time. When the agent is close to the steady state, the difference in rewards brought by different actions is very small. As a terminal reward, $r_{4}$ can prevent the agent from fluctuating near the steady state and improve sample efficiency. Incorporating $r_{3}$ allows the optimal strategy for oscillation suppression to be adjustable, effectively circumventing the issue of excessively prolonged adjustment times. Nonetheless, the value of $r_{3}$ must be judiciously selected; if $r_{3}$ is too large, the frequency deviation will become larger, resulting in poorer oscillation suppression effect. Conversely, should the value be excessively minimal, its efficacy in application will be compromised.

### 3.2. Principle of Soft Actor Critic

**Figure 5:**
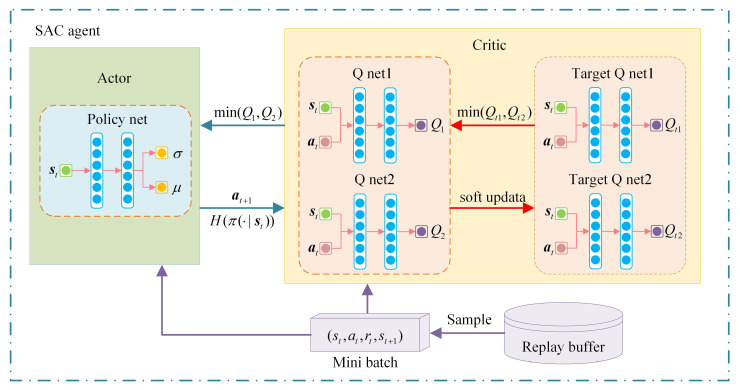
The framework of the SAC algorithm.

This paper employs the soft actor critic(SAC) algorithm to address the MDP associated with the optimization of the VSG dynamic response. SAC is a model-free and offline RL algorithm. By incorporating the concept of maximum entropy, it not only enhances the algorithm’s robustness but also improves the agent’s exploration capability, thereby accelerating the learning speed [34,35]. Figure 5 shows the framework of the SAC algorithm.

The learning objective of SAC extends beyond maximizing the cumulative rewards, it also aims to maximize the entropy of each state [36,37,38]. Therefore, the objective function of SAC is as follows:

(22) $$L\left(\pi \right)=\sum_{t=0}^{\infty }E_{\left(s_{t},a_{t}\right)∼\rho_{\pi }}\left[r\left(s_{t},a_{t}\right)+\alpha H\left(\pi \left(\cdot |s_{t}\right)\right)\right]$$

where $\rho_{\pi }$ represents the state transition equation; π represents the policy function; α is the temperature coefficient, delineating the relative significance of entropy terms in comparison to reward terms. It plays a crucial role in modulating the level of randomness in the policy; *H* represents the entropy of policy π in state $s_{t}$ and is given by

(23) $$H\left(\pi \left(\cdot |s_{t}\right)\right)=-\log\left(\pi \left(a_{t}|s_{t}\right)\right)$$

RL algorithms use the Q-value to represent the expected cumulative reward that can be obtained by taking action $a_{t}$ in state $s_{t}$. Different from general value-based RL algorithms, the Q-value in SAC contains both the reward value and action entropy. According to the Bellman equation, the soft Q-value can be calculated as follows:

(24) $$Q\left(s_{t},a_{t}\right)=r\left(s_{t},a_{t}\right)+\gamma E_{s_{t+1}∼p}[V\left(s_{t+1}\right]$$

where

(25) $$V\left(s_{t}\right)=E_{a_{t}∼\pi }\left[Q\left(s_{t},a_{t}\right)-\alpha \log\pi \left(a_{t}|s_{t}\right)\right]$$

is the soft state value function.

The SAC algorithm comprises a total of five neural networks, including a policy network, two critic networks and two target critic networks. The role of the critic network is to approximate the soft Q-value. Employing dual critic networks helps mitigate the issue of overestimating the Q-value. The parameters of the soft Q-function can be trained by minimizing the soft Bellman residual, which is expressed as follows:

(26) $$L_{Q}\left(\theta \right)=E_{(s_{t},a_{t})∼D}\left[\frac{1}{2}{\left(Q_{\theta }\left(s_{t},a_{t}\right)-r\left(s_{t},a_{t}\right)-\gamma E_{s_{t+1}∼\rho_{\pi }}\left[V\left(s_{t+1}\right)\right]\right)}^{2}\right]$$

where θ is the parameter of critic network; D represents the experience replay buffer.

The policy network outputs the probability distribution of the action. This paper uses Gaussian probability distribution, so the policy network outputs the mean and standard deviation of the Gaussian probability distribution. The loss function for the policy network is as follows:

(27) $$L_{\pi }\left(\phi \right)=E_{s_{t}∼D}\left[E_{a_{t}∼\pi_{\phi }}\left[\alpha \log\left(\pi_{\phi }\left(a_{t}|s_{t}\right)\right)-Q_{\theta }\left(s_{t},a_{t}\right)\right]\right]$$

where ϕ represents the parameter of the policy network.

During training, SAC maintains a balance between exploration and exploitation by adjusting the size of temperature coefficient α. Larger temperature coefficients correspond to increased exploration, while smaller coefficients correspond to heightened exploitation. However, determining the appropriate temperature coefficient can be challenging. In order to solve this problem, the SAC algorithm sets a target entropy $\bar{H}$ for each task and automatically updates the temperature coefficient during the training process. The loss function of temperature coefficient is designed as follows:

(28) $$L\left(\alpha \right)=E_{a_{t}∼\pi_{t}}\left[-\alpha \log\left(\pi_{t}\left(a_{t}|s_{t}\right)\right)-\alpha \bar{H}\right]$$

The parameters of each neural network are updated through the gradient descent method:

(29) $$\left\{\begin{array}{c} \theta_{i}\leftarrow \theta_{i}-\lambda_{Q}{\hat{\nabla }}_{\theta_{i}}L_{Q}\left(\theta_{i}\right)\;i\in 1,2\\ \phi \leftarrow \phi -\lambda_{\pi }{\hat{\nabla }}_{\phi }L_{\pi }\left(\phi \right)\\ \alpha \leftarrow \alpha -\lambda_{\alpha }{\hat{\nabla }}_{\alpha }L\left(\alpha \right) \end{array}\right.$$

where ${\hat{\nabla }}_{\theta_{i}}L_{Q}\left(\theta_{i}\right)$, ${\hat{\nabla }}_{\phi }L_{\pi }\left(\phi \right)$ and ${\hat{\nabla }}_{\alpha }L\left(\alpha \right)$ represent the gradients of critic network, policy network and temperature coefficient, and $\lambda_{Q}$, $\lambda_{\pi }$ and $\lambda_{\alpha }$ represent the corresponding learning rate, respectively.

## 4. Simulation Results

To assess the effectiveness and feasibility of the VSG parameter adaptive control strategy based on SAC proposed in this paper, a single grid-connected VSG simulation system shown in Figure 1 is constructed using Simulink. The key parameters of the simulation system and RL agent are detailed in Table 1.

**Figure 6:**
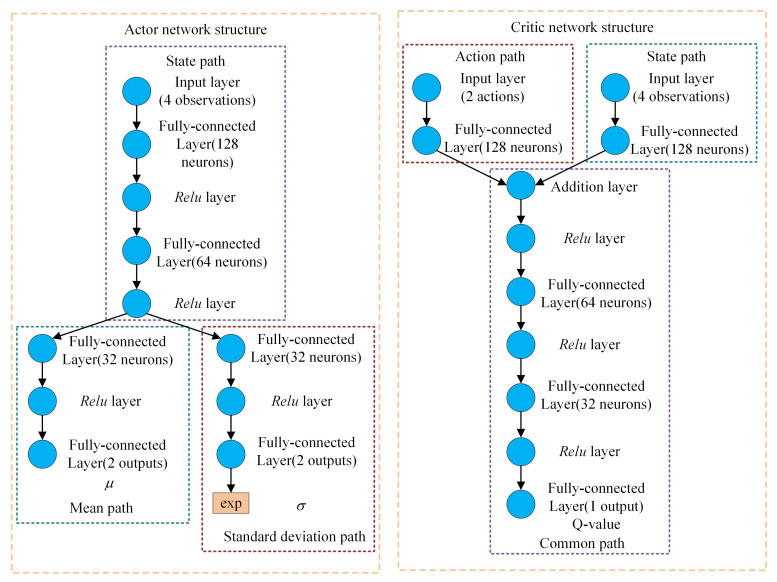
Structures of the actor and critic network.

The RL agent is developed utilizing the RL toolbox in MATLAB, with the architecture of the actor network and the critic network illustrated in Figure 6. In this article, a Gaussian distribution is utilized as the action probability distribution. As depicted in Figure 6, the state information initially traverses through the state pathway, subsequently splitting into two branches. One branch is responsible for outputting the mean μ of the Gaussian distribution, while the other branch outputs the standard deviation σ. The role of the exponential function (exp) here is to ensure that the output value of the standard deviation remains positive. The action is derived by randomly sampling from the Gaussian distribution $N(\mu ,\sigma^{2})$. Given that the sampling process is non-differentiable, to facilitate the actor in executing the neural network’s backpropagation process, the following formula is employed in practical applications to derive the action:

(30) $$a_{t}=\tanh\left(\mu +\sigma \cdot \epsilon_{t}\right),\;\epsilon_{t}∼N\left(0,1\right)$$

where $\epsilon_{t}$ is random noise obeying the standard Gaussian distribution. The value range of the tanh function is from −1 to 1, serving to transform Gaussian distribution sampling, which possesses an infinite value range, into a finite action value. The critic network is composed of three segments: the action path, the state path and the common path. The action path receives action information as its input, while the state path is fed with state information. These two paths converge on the common path, which subsequently processes the combined information to output the Q-value. The activation functions utilized in the neural networks are all *Relu* functions, as represented by the following formula:

(31) Relu(x)=max(0,x)

To enhance the generalization ability of reinforcement learning agents, random disturbances are introduced to the reference active power and load power during the training process. The range of variation for the reference active power is 0 kW to 50 kW, and for load power, it is 5 kW to 30 kW.

### 4.1. Comparison of Different Reward Functions

The optimal strategy that the agent ultimately secures is determined by the structure of the reward function. To assess the impact of the setting time penalty term introduced in this article, we compare the training outcomes derived from various reward functions. The RL algorithm utilized in all cases is the SAC algorithm. There are three types of reward functions: The reward function of Case 1 includes only the oscillation suppression rewards $r_{1}$, $r_{2}$ and the terminal reward $r_{4}$, excluding the setting time penalty term $r_{3}$. This reward function is designed to suppress power and frequency oscillations to the greatest extent possible, but it may result in excessively prolonged setting times. The reward function for Case 2 encompasses all components, yet the setting time penalty term $r_{3}$ is prioritized with a larger proportion. This reward function expects a rapid system response rate while overlooking the optimization of the process. In Case 3, the reward function also comprises all elements, but it maintains a balanced proportion of $r_{1}$, $r_{2}$ and $r_{3}$, aiming to achieve equilibrium between process optimization and adjustment time.

**Figure 7:**
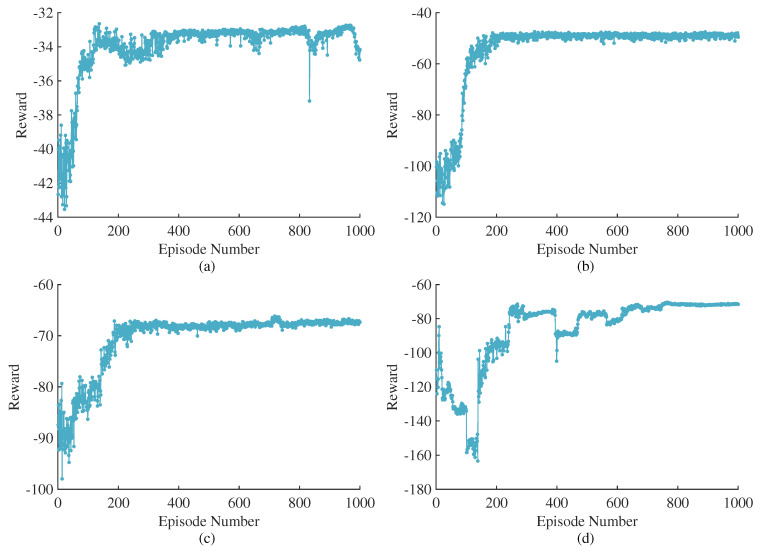
Training results with different reward functions and RL algorithms. (**a**–**c**) are training results of SAC with different reward functions, and (**d**) is training results of DDPG with Case 3. (**a**) Case 1. (**b**) Case 2. (**c**) Case 3. (**d**) DDPG with Case 3.

**Figure 8:**
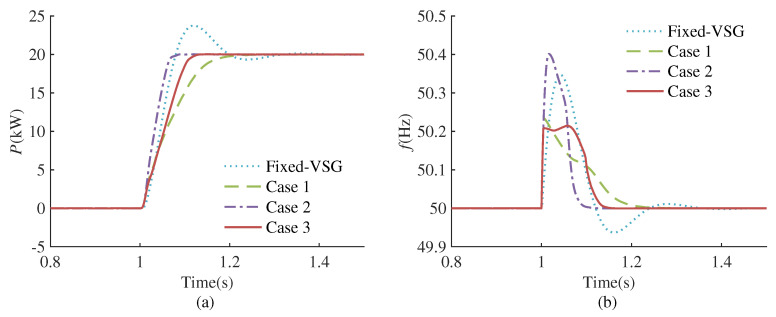
Simulation results with different reward functions. (**a**) Active power response. (**b**) Frequency response.

Figure 7a–c shows the training results of different reward functions. Under a range of reward functions, the cumulative rewards demonstrate convergence, suggesting that the agents have successfully identified their respective optimal strategies. Next, we conduct time-domain simulations for the trained agents with various reward functions in Simulink. The simulation scenario involves applying a step disturbance that changes the active power reference value from 0 kW to 20 kW at 1.0 s. The simulation results are depicted in Figure 8.

As observed in Figure 8, compared with the traditional VSG with fixed parameters (Fixed-VSG), there are no secondary oscillations in the active power and frequency in all three cases. This outcome is attributed to the inclusion of $r_{1}$ and $r_{2}$ in the reward function. The introduction of the setting time component alters the agent’s optimal strategy. An increase in the proportion of the setting time penalty term results in a gradual reduction in the setting time of the system. Further analysis reveals that since the reward function of Case 1 lacks the $r_{3}$ term, it more effectively suppresses the maximum frequency deviation, yet the optimization of the setting time is not pronounced. The reward function of Case 2 incorporates $r_{3}$ with a substantial weighting, while this significantly reduces the setting time, it leads to an increased frequency deviation and results in a deterioration of the frequency response. Case 3 establishes a balanced ratio among the various rewards, effectively suppressing the maximum frequency deviation and considerably reducing the setting time, aligning closely with the desired optimization objectives. This proves the effectiveness and flexibility of the method proposed in this paper.

### 4.2. Comparison of Different RL Algorithms

Furthermore, to ascertain the superiority of the SAC algorithm, this article employs the DDPG algorithm, which also operates under the Actor–Critic framework, training the agent using the reward function specified in Case 3. To maintain fairness in the comparison, the shared structures and parameters across each agent are initialized with identical values. For instance, the critic networks of SAC and DDPG are configured with the same architecture, and the initial values of the neural networks are also aligned. Considering that SAC employs a stochastic policy, while DDPG utilizes deterministic policies, the pathway from input to mean output in the SAC’s actor network is identical to the actor networks in DDPG. Moreover, the different algorithms also share the same learning rate and soft update coefficient. The reward curve of DDPG is shown in Figure 7d.

Comparing Figure 7c,d, it is apparent that SAC converges to the optimal strategy in just 200 rounds, whereas DDPG takes up to 750 rounds to converge, signifying a faster learning pace for SAC. The reward curve of SAC remains smooth throughout the learning process, contrasting with the large fluctuations observed in DDPG’s reward curve, which suggests a more stable learning trajectory for SAC. SAC’s cumulative reward ultimately settles at −67.4, while DDPG’s reward converges at −71.6, further evidencing the superior learning effectiveness of SAC. This conclusively demonstrates the superiority of the SAC algorithm.

### 4.3. Case Studies

In this subsection, our purpose is to verify the effectiveness of the well-trained VSG controller and compare its control effect with other methods in different scenarios. The proposed method (SAC-VSG) is trained using the balanced reward function specified in Case 3. The simulation scenarios are primarily executed under three distinct disturbance conditions: active power reference disturbance, load power disturbance and grid frequency disturbance. The methods included in the comparison are traditional fixed parameter control (Fixed-VSG), linear adaptive control (Adaptive-VSG) as mentioned in [9], fuzzy adaptive control (Fuzzy-VSG) from [10], and DDPG adaptive control (DDPG-VSG) utilizing the reward function from Case 3.

#### 4.3.1. Active Power Reference Disturbance

**Figure 9:**
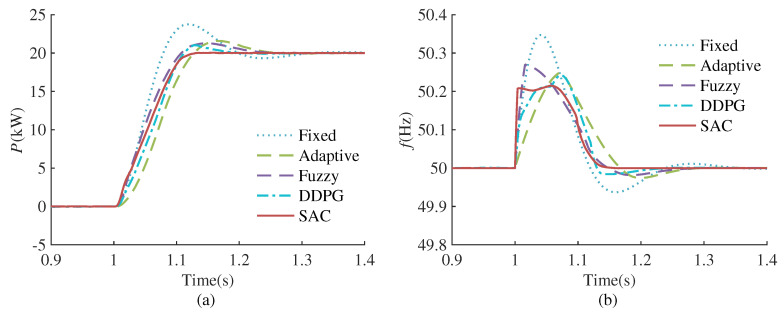
Performance comparison of different VSG controllers under reference active power change. (**a**) Active power response. (**b**) Frequency response.

This case study compares the control effects of different methods in response to a step disturbance in the reference active power. The simulation test conditions are as follows: Initially, the system is in a stable state, and the reference active power by VSG is set to 0 kW. At 1.0 s, there is a step change for the reference active power from 0 kw to 20 kW. The simulation results are shown in Figure 9. We evaluate different control methods based on active power overshoot σ (the steady-state range is set to ±1%), maximum frequency deviation Δf and setting time $t_{s}$ presented in Table 2.

It can be seen that when the reference active power changes, the control effect of Fixed-VSG is not ideal, and there is a large oscillation in both active power and frequency response. Adaptive-VSG and Fuzzy-VSG can improve the small signal oscillation problem of the traditional VSG control to a certain extent, but the suppression of frequency fluctuations and the shortening of setting time are not obvious, and while the DDPG algorithm can further optimize overshoot, maximum frequency deviation, and setting time, there remains scope for enhancement. However, SAC-VSG has almost no active power overshoot, the maximum frequency deviation is also very well suppressed, and the setting time is greatly shortened. It can be concluded that the optimization effect of the proposed method is better than for the other methods in all aspects under the reference active power change.

#### 4.3.2. Load Disturbance

**Figure 10:**
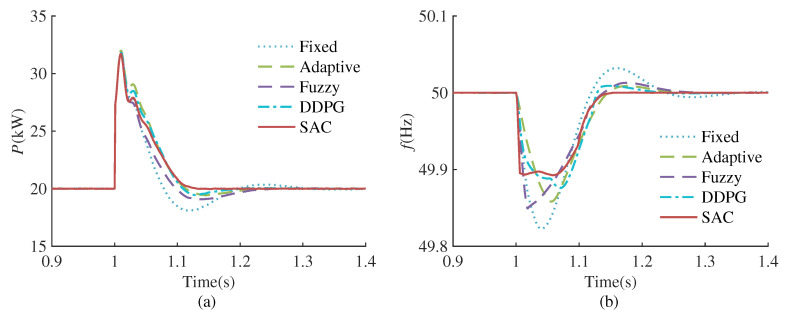
Performance comparison of different VSG controllers under load change. (**a**) Active power response. (**b**) Frequency response.

This case study compares the control effects of different methods under load disturbances. The simulation test conditions are as follows: At the initial moment, the system is in a stable state, with the active power of the load set at 10 kW. At 1.0 s, a load with active power of 20 kW is connected. The simulation results are shown in Figure 10, and the various performance indicators of different control methods are presented in Table 3.

Figure 10 shows that when the load active power changes suddenly, in order to satisfy the power balance, the inverter output power first changes with the load power and then gradually returns to the reference. Compared to the other four methods, SAC-VSG can restore to the equilibrium state more quickly and stably during power restoration. It can be concluded from Table 3 that SAC-VSG is also better than the other four methods in terms of active power overshoot, maximum frequency deviation and setting time under load power disturbance.

#### 4.3.3. Grid Frequency Disturbance

**Figure 11:**
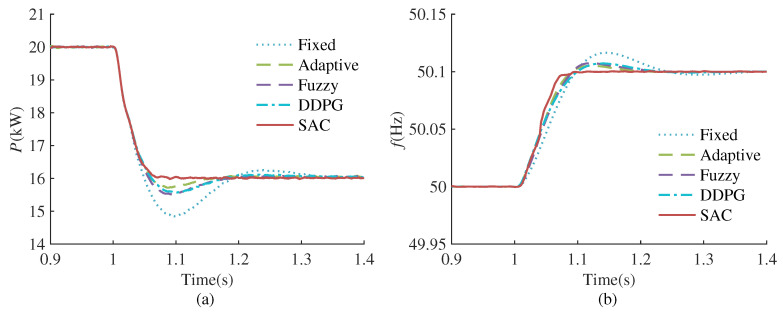
Performance comparison of different VSG controllers under grid frequency change. (**a**) Active power response. (**b**) Frequency response.

In order to further verify the robustness of the proposed method, this case simulates and compares the control effects of different methods when small disturbances occur in the grid frequency. The simulation test conditions are as follows: The system is in a stable state at the initial moment, with the reference active power is 20 kW. At 1.0 s, the grid frequency changes from 50 Hz to 50.1 Hz. The comparison of simulation results is shown in Figure 11.

As shown in Figure 11, when the grid frequency undergoes changes, there exists a static deviation between the output active power and reference active power, quantified as $\Delta P=-D_{0}\omega_{0}\Delta \omega_{g}$, where $D_{0}$ denotes the steady-state damping coefficient, set to 20 in this case. Consequently, a 0.1 Hz increase in grid frequency results in a 4 kW decrease in reference active power, and vice versa, an increase of 4 kW occurs. Figure 11 shows that the suppression effect of frequency oscillation and active power oscillation of SAC-VSG is more obvious than the other four methods, indicating that the proposed method is also suitable for disturbances in the power grid frequency.

## 5. Conclusions

Based on VSG control, this paper proposes an adaptive control strategy for virtual inertia and damping coefficient based on SAC to address the oscillation problem existing in the dynamic process of grid-connected VSG control. In order to better achieve the expected control performance, the oscillation suppression problem of VSG is described as a multi-objective optimization problem, and the optimization of frequency, active power and setting time is comprehensively considered. The problem is then transformed into a RL task and solved using the SAC algorithm. RL algorithms learn strategies through interaction with the environment and can achieve optimization in different scenarios. Compared with existing VSG adaptive control methods, the proposed method has better optimization effects in various application scenarios. Moreover, the proposed method does not require expert knowledge and system mathematical models and is a fully automatic optimization algorithm.

The RL parameter adaptive adjustment algorithm in this paper currently only considers the VSG control of a single inverter and can conduct research on multi-machine parallel systems in the future.

## Figures and Tables

**Table 1:** Key parameters of simulation model.

Parameters	Value	Parameters	Value
$U_{dc}$	750V	$\omega_{0}$	314rad/s
$U_{0}$	220V	BatchSize	128
$L_{f}$	3.2mH	ReplayBuffer	100,000
$C_{f}$	20μF	$\lambda_{Q}$	0.001
$R_{f}$	0.1Ω	$\lambda_{\pi }$	0.001
$L_{g}$	3.2mH	$\lambda_{\alpha }$	0.001
$R_{g}$	0.1Ω	γ	0.99

**Table 2:** Performance indicators under reference active power disturbance.

Methods	σ%	Δf/Hz	$t_{s}/s$
Fixed	18.7	0.35	0.30
Adaptive	8.9	0.25	0.26
Fuzzy	6.2	0.27	0.25
DDPG	5.1	0.24	0.20
SAC	0.0	0.21	0.12

**Table 3:** Performance indicators under load power disturbance.

Methods	σ%	Δf/Hz	$t_{s}/s$
Fixed	19.2	0.18	0.30
Adaptive	5.8	0.14	0.26
Fuzzy	9.2	0.15	0.25
DDPG	5.5	0.12	0.19
SAC	0.0	0.11	0.12

## Data Availability

Data are contained within the article.
